# Nanoliposome-Encapsulated and Non-Encapsulated Phenolics From *Achillea millefolium* and Their Biological Function in Mice Challenged by *Campylobacter jejuni*: A Comparative Study

**DOI:** 10.3389/fmolb.2021.832022

**Published:** 2022-02-02

**Authors:** Nikta Nateghi, Ehsan Karimi, Ehsan Oskoueian

**Affiliations:** ^1^ Department of Biology, Mashhad Branch, Islamic Azad University, Mashhad, Iran; ^2^ Department of Research and Development, Arka Industrial Cluster, Mashhad, Iran

**Keywords:** phytobiotic, phytogenics, antibiotic alternative, target delivery, gene expression

## Abstract

The objective of this research was to develop the nanoliposome-encapsulated phenolic rich fraction from *Achillea millefolium* (*A. millefolium*) and to investigate its antibacterial and health-promoting activities in mice challenged by pathogenic foodborne *Campylobacter jejuni*. The *A. millefolium* was extracted and the ethyl acetate fraction was found to be the phenolic-rich fraction (PRF) containing 14.72 ± 2.39 mg gallic acid equivalent (GAE)/g dry weight (DM). Base on the results, the synthesized nanoliposome-loaded PRF (PRF-NLs) with the size of 187.2 nm exhibited homogeneous dispersion (PDI 0.213) and moderate stability behavior in colloidal dispersions (Zeta potential −37.45). The non-encapsulated PRF and PRF-NLs were gavaged orally in the mice for 28 days, and mice were challenged with *C. jejuni* on day 21. The results indicated that the dietary supplementation of non-encapsulated PRF and PRF-NLs significantly (*p* < 0.05) improved the average daily weight gain, food intake, liver function, antioxidant status, and morphostructural characteristics of the ileum. However, the PRF-NLs appeared to be more potent as compared to non-encapsulated PRF. The higher biological activity of PRF-NLs could be associated with the higher intestinal solubility and absorption of nanoliposome-encapsulated PRF. Thereby, the nanoliposome-encapsulated PRF could be considered as a natural antibiotic alternative called phytobiotic to prevent intestinal infection caused by enteropathogenic *C. jejuni.*

## Introduction

*Campylobacter jejuni* (*C. jejuni*), a Gram-negative bacterium that is known as a food-borne pathogen (Chlebicz and Śliżewska 2018). The natural reservoirs of *C. jejuni* are the wild bird’s intestine, where is a suitable niche for its dissemination and survival (Abdollahpour et al., 2015). It mediates the N-linked glycosylation of over 30 colonizing, adherence, and invasion proteins (Dasti et al., 2010; Lai et al., 2013). Since this pathogenic bacterium has been more prevalent worldwide and has become increasingly resistant to antibiotics therefore, alternative natural antimicrobials are vital to inhibit or decrease their contamination in food and food-producing animals (Marotta et al., 2019).

The *Achillea millefolium* L. (*A. millefolium*) (known as yarrow) is a well known medicinal plant that belongs to the Asteraceae families and contains more than 100 species (Dias et al., 2013). This herbal medicine is famous in applied medicine, pharmacological studies, and antibacterial researches due to its important natural bioactive compounds including salicylic and succinic acids, folic acid, caffeic acid, kaempferol, luteolin, apigenin, and other phenolic and flavonoid compounds. *A. millefolium* is wildly distributed in Asia, Africa, Europe, and America and cultivated in various regions all around the world (Dias et al., 2013; Tadić et al., 2017). It also indicated strong cytotoxic and antimicrobial properties according to the high content of phenolic compounds (Taşkın et al., 2016; Abou Baker 2020).

Polyphenols are natural bioactive compounds that attracted a good deal of attention due to scavenging the free radicals through donating hydrogen atoms and interrupting chain oxidation reaction. Several experiments worldwide have evaluated and proven their biological properties including effectiveness against various pathogenic bacteria. There are over 9000 phenolic compounds that have been extracted (Wang et al., 2011) mainly from dietary supplements including apples, tea, red wine, tomatoes, onions and exhibit strong antioxidant and antimicrobial potential (Wang et al., 2018).

However, the widespread use of phenolic compounds has been limited due to their low solubility resulting in poor absorption and biological activities. Previous studies revealed that different carriers like liposomes could improve solubility and enhance the bioactivity of these natural components (Rafiee et al., 2017). Nanoliposomes consist of phospholipid bilayers that are microscopic carriers that can control the release of natural bioactive compounds like polyphenols to the target place and enhance the effectiveness and cellular uptake of the encapsulated natural constitute (Hasan et al., 2014). One of the great benefits of applying nanoliposomes is the acquired features when added to bioactive compounds as their physicochemical potentials including size which enhance their solubility and bioavailability. Meanwhile, they have low toxicity and high biocompatibility to capsulate both hydrophilic and lipophilic active components (Figueroa-Robles et al., 2021). The objective of this research was to develop the nanoliposome-encapsulated phenolic rich fraction from *Achillea millefolium* and to investigate its antibacterial and health-promoting activities in mice challenged by pathogenic foodborne *C. jejuni*.

## Materials and Methods

### Plant Material and Reagents

Herbal medicine markets in Mashhad, Iran, provided fresh aerial parts of *A. millefolium*. Sigma Aldrich (Germany) supplied soybean lecithin with a purity of 99%. As a food-borne pathogen, *C. jejuni* (RTCC 1097) was provided from Razi Vaccine and Serum Research Institute, Karaj, Iran (Gharib Naseri et al., 2012; Ebrahimi et al., 2016). As part of the gene expression analysis, the RNeasy Mini kit from Qiagen (Hilden, Germany), SYBR Green PCR Master Mix from Qiagen (Hilden, Germany), cDNA Quantitect Reverse Transcription Kit (Qiagen, Hilden, Germany), and SYBR Green PCR Master Mix from Qiagen (Hilden, Germany) were utilised. In this study, DNA was extracted using the QIAamp DNA Stool Mini Kit from Qiagen, Hilden, Germany. Besides these, Merck (Germany) provided the rest of the reagents.

### Fractionation and Total Phenolic Determination

We cleaned, separated, and freeze dried the freshly cut aerial parts of *A. millefolium* in the first step. Using the reflux method, 900 ml aqueous methanol (80% v/v) and 100 ml of 6 M HCl were added to 100 g of the dried powder for 2 h (Karimi et al., 2019) to do the extraction. An electronic rotary evaporator (Buchi, Flawil, Switzerland) was then used to evaporate the extract (60°C). A separating funnel and different solvents (hexane, ethyl acetate, n-butanol, chloroform, and water) were used to fractionate the dried aqueous-methanolic extract (Oskoueian et al., 2020). A vacuumed rotary evaporator was used to filter and concentrate the supernatant after fractionation. In a test tube covered with aluminum foil, 0.1 ml of the extract was mixed with 2.5 ml of Folin-Ciocalteu reagent (1:10 v/v) and 2 ml of 7.5% sodium carbonate to measure the total phenolic compounds in each fraction. Vortexing of the test tubes and measuring absorbance at 765 nm were done (Oskoueian et al., 2020). These results are reported in milligrams of gallic acid equivalents (GAE) per Gram dry weight. Phenolic rich fractions (PRFs) are defined as fractions containing the most phenolics.

### Nanoliposomes Preparation

In 196 g of hot distilled water, 4 g of lecithin were hydrated and agitated for 2 h. To reach the final concentration of 2000 ppm, the PRF after dissolving in ethanol was gradually added to the mixture (2 h) and stirred. The nanoliposome-encapsulated phenolic rich fraction (PRF-NLs) that was obtained after sonication (Cole-Parmer Instrument, United States) for 4–6 min was applied for further characterization.

### Characterization of Nanoliposomes

Nanoliposome characteristics such as particle size and zeta potential, were evaluated with Zetasizer Nano ZS-Malvern. The Shape and Nanoliposomes Size Dimensions were also confirmed on the basis of Field Emission Scanning Electron Microscopy (FESEM). According to the fractionation section (Oskoueian et al., 2020), the total phenolic content of nanoliposomes was determined.

### Phenolic Profiling of Nanoliposomes

Reversed-Phase High-Performance Liquid Chromatography (RP-HPLC) was used to identify the phenolic compounds present in the nanoliposomes-loaded PRF from *A. millefolium*. In the present experiment, the phenolic standards included catechin, vanillic acid, naringin, gallic acid, syringic acid, salicylic acid, caffeic acid, pyrogallol, cinnamic acid, ellagic acid, chrysin, and ferulic acid. There are two solvents used here, deionized water (solvent A) and acetonitrile (solvent B). After eluting and equilibrating the column with 85 percent solvent A and 15 percent solvent B, the injection was performed. Following 60 min, the ratio of solvent B was increased to 85%. After 5 min of running the experiment, the ratio of solvent B had decreased to 15 percent. A flow rate of 1 ml/min was used for the next analysis with a ratio of 70 min. The analysis of phenolic at 280 nm was conducted with an analytical column (Intersil ODS-3 5um 4.6 mm × 150 mm Gl Science Inc. United States).

### Bacterial Preparation

This experimental challenge was designed to determine the pathogenicity of *C. jejuni*, which was isolated from the cloaca of commercial broiler chicken (Gharib Naseri et al., 2012; Ebrahimi et al., 2016). The *C. jejuni* was propagated in microaerophilic conditions at 42°C using selective culture media (Oxid, United Kingdom). A specific viable concentration of 1 × 10^9^ cfu/ml was obtained by harvesting the bacteria and diluting them in peptone water. Prior to oral gavage, the inoculum was maintained on ice for less than 1 h.

### Animal Trial

At the Razi Vaccine and Serum Research Institute in Mashhad, we purchased 40 white male Balb/c mice (20–25 g). To adapt to lab conditions, the mice were kept in individual cages at 58% humidity, 10% humidity, 23 °C, and 1% temperature with 12-h periods of light and darkness for 7 days. During the experiment, 40 mice were divided into four groups of ten and provided with a standard pelleted diet and tap water (Javaneh Khorasan, Mashhad, Iran). There were four experimental groups:

T1: normal diet.
T2: normal diet + infected by *C. jejuni* on day 21.
T3: Normal diet enriched by PRF (10 mg TPC/kg BW/day) + infected by *C. jejuni* on day 21.
T4: Normal diet enriched by nanoliposome-encapsulated PRF (10 mg TPC/kg BW/day) + infected by *C. jejuni* on day 21.

All mice underwent experimental treatment for 4 weeks, and the oral infection (108 cfu of *C. jejuni*) was administered once on day 21 of treatment. General health and food consumption were monitored daily. The mice were sacrificed at the day 28th of the experiment with pentobarbital-HCL (50 mg/kg, i. p.). We collected blood, ileum samples, and liver samples immediately for gene expression analysis, liver enzyme analysis, morphometric evaluation of the ileum, and lipid peroxidation assay. In the experiment, mice were weighed twice, once at the beginning, and once at the end. A code of ethics was adopted by the Islamic Azad University of Mashhad with regards to the animal experiments, IR. IAU.MSHD.REC.1399.013.

### Liver Enzymes and Lipid Peroxidation Assay

A blood auto-analyzer (Hitachi 902, Japan) was used to determine serum levels of liver enzymes, such as ALT, AST, and ALP. Peroxidation of lipids in the liver tissue was measured as previously described by Shafaei et al. (2020). Using liver tissue homogenate, 200 µl of lysate was mixed respectively with distilled water (300 µl), BHT (35 µl), sodium dodecyl sulfate (165 µl), and thiobarbituric acid (2 µl). In the following step, the heated solution was cooled for 60 min, then mixed with 2 ml of n-butanol, and centrifuged at 2000 ×g for 5 min. At 532 nm, the absorbance of the n-butanol part of the reaction was measured and the results were expressed as the percentage of malondialdehyde (MDA) variations relative to a control sample.

### Histopathology and Morphometric Analyses

At the end of the *in vivo* experiment, the mice were sacrificed and the liver, kidney, and ileum were separated and washed using the physiological serum. They were fixed in buffered formalin (10% formalin in 0.1 M sodium phosphate buffer, pH7), paraffinized, sliced, and stained according to the hematoxylin/eosin protocol (Shafaei et al., 2020). The histopathology slides were observed under a light microscope using a magnification of 20×. The morpho-structural characteristics of ileum including villus height, villus width, crypt depth, and goblet cell count were determined (Navarrete et al., 2015).

### Gene Expression Analysis

In order to study how ileum tissue responded to different treatments, we determined the expression of major inflammation markers such as inducible nitric oxide synthase (iNOS), cyclooxygenase 2 (COX2), and antioxidant biomarkers like superoxide dismutase (SOD) and glutathione peroxidase (GPx). RNA was extracted from freshly frozen ileum tissues using an RNeasy Mini kit (Qiagen, Hilden, Germany) after they had been crushed and prepared in accordance with the recommended protocol. Qiagen, Hilden, Germany) reverse transcription kit was used to make the cDNA. Table 1 shows the characteristics of the primers for the key genes and the housekeeping gene (Beta-actin). In a comparative Real-time PCR (Roche Diagnostics), Qiagen’s SYBR Green PCR Master Mix was used (Hilden, Germany). We amplified the target genes using the following protocol: for initial denaturation, 95°C for 5 min (1X), followed by 35 cycles of 95°C for the 30 s, primer annealing at 60 and 58 for 30 s for the inflammatory genes (COX2, iNOS), and antioxidant genes (SOD, GPx), respectively and 30 s of extension in 72°C. In this study, beta-actin was used as a reference gene and the respective gene expressions were normalized to those of the control group (Kathirvel et al., 2010).

**TABLE 1:** The primer sets characteristics used in this study.

Gene	Forward (5′ →3′)	Reverse (5′ →3′)	References
COX2	caa​gca​gtg​gca​aag​gcc​tcc​a	ggc​act​tgc​att​gat​ggt​ggc​t	Jain et al. (2008)
iNOS	cac​ctt​gga​gtt​cac​cca​gt	acc​act​cgt​act​tgg​gat​gc	Kou et al. (2011)
SOD	gag​acc​tgg​gca​atg​tga​ct	gtt​tac​tgc​gca​atc​cca​at	Kathirvel et al. (2010)
GPx	gtc​cac​cgt​gta​tgc​ctt​ct	tct​gca​gat​cgt​tca​tct​cg	Kathirvel et al. (2010)
β-actin	cct​gaa​ccc​taa​ggc​caa​cc	cag​ctg​tgg​tgg​tga​agc​tg	Shafaei et al. (2020)

### *Campylobacter jejuni* Population Analysis

A real-time PCR (LightCycler 96 instrument, Roche, Basel, Switzerland) was used to determine the relative abundance of the colonized *C. jejuni* population in the ileum digesta. As the ileum is the main site of enteropathogens’ fermentation, propagation, and colonization the monogastric, the population of *C. jejuni* in this study was only examined in the ileum section. Initial denaturation was performed at 95°C for 5 min (1X), followed by 35 cycles of 95°C for 30 s, followed by primer annealing at 60, and then 55 for 25 s for *C. jejuni* and total bacteria, respectively, and 20 s extension in 72°C. Table 2 shows the characteristics of primers. DNA was extracted from ileum digesta using a QIAamp DNA Stool extraction kit (QIAGEN, Germany). This study used the SYBR GREEN Master Mix (BIO FACT, Korea). A quantitative real-time PCR assay was performed using previously published primers. In this study, we used the ∆∆ Ct method to compare fold changes in the *C. jejuni* bacteria population and expressed the results as fold changes in the amount of *C. jejuni* relative to the overall bacteria population (Si et al., 2007; Feng et al., 2010).

**TABLE 2:** The list of the primers used for ileum microbial population analysis.

Bacteria	Forward (5′ →3′)		Reverse (5′ →3′)	References	
*C. jejuni*	cgg​gat​agt​tat​agt​att​gaa​gtt	gaa​gga​gca​taa​tag​gat​ctt​g			Razzuoli et al. (2018)
Total bacteria	cggcaacgagcgcaaccc	cca​ttg​tag​cac​gtg​tgt​agc​c			Denman and McSweeney (2006)

### Statistical Analysis

This study used the general linear model (GLM) of SAS (Version 9.1) to analyze the collected data. The significance of the difference among treatments was determined using Duncan’s multiple range test. To determine whether there were statistically significant differences among treatments, a p-value of <0.05 was used. The experiments were conducted in triplicate and the results were presented as mean values ±SE of the mean or standard deviation.

## Results and Discussion

### Fractionation

The total phenolic content of the fractions are as follow: ethyl acetate fraction 14.72 ± 2.39> n-butanol fraction 12.1 ± 1.19 > chloroform fraction 10.5 ± 0.73 > hexane fraction 8.6 ± 0.49 > water fraction 3.56 ± 0.51 mg GAE/g DM, respectively. Hence, the ethyl acetate fraction was chosen as a phenolic-rich fraction (PRF) and this fraction was used for further evaluations.

### Characterization of PRF-NLs

**FIGURE 1:**
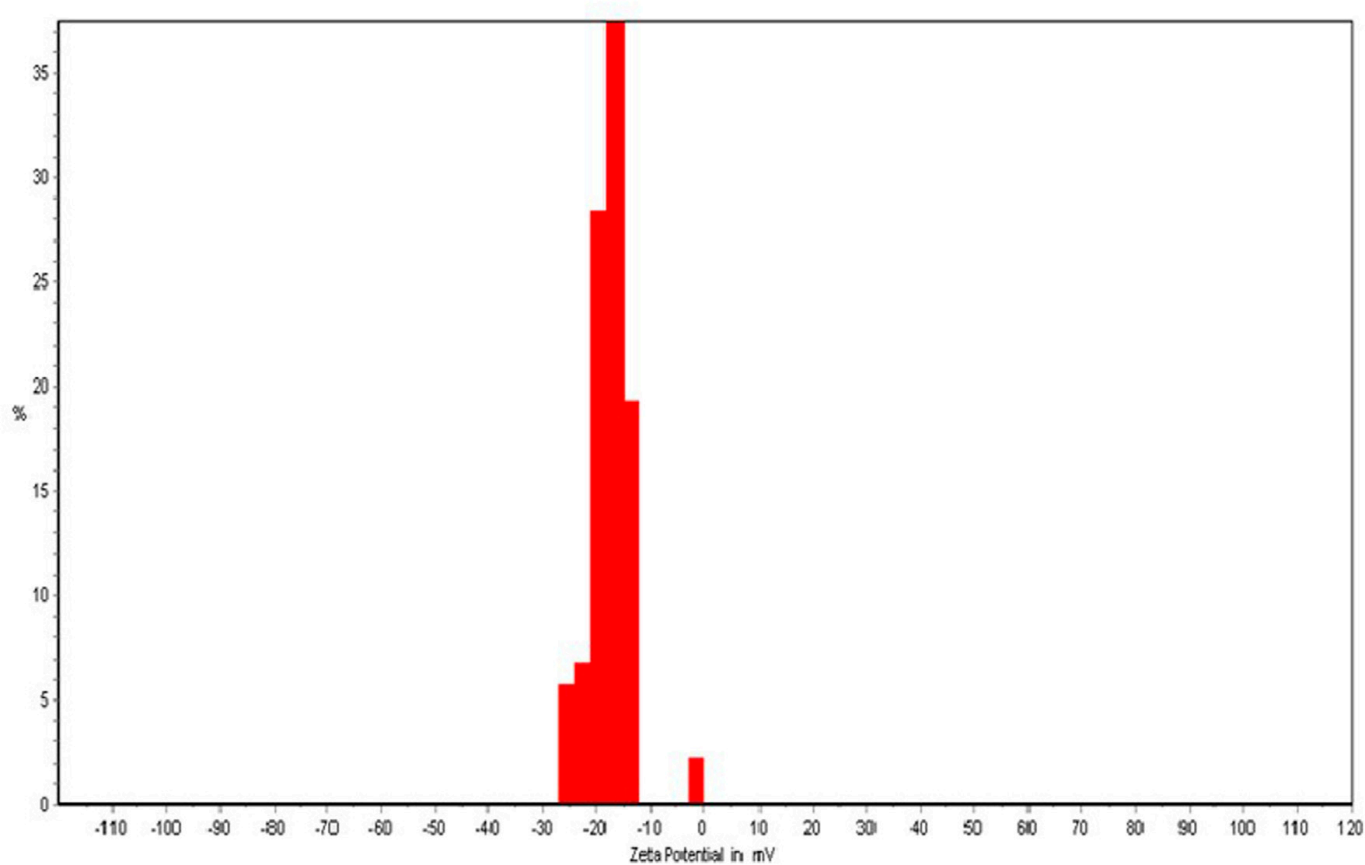
The zeta potential of *Achillea millefolium* phenolics-loaded nanoliposomes.

**FIGURE 2:**
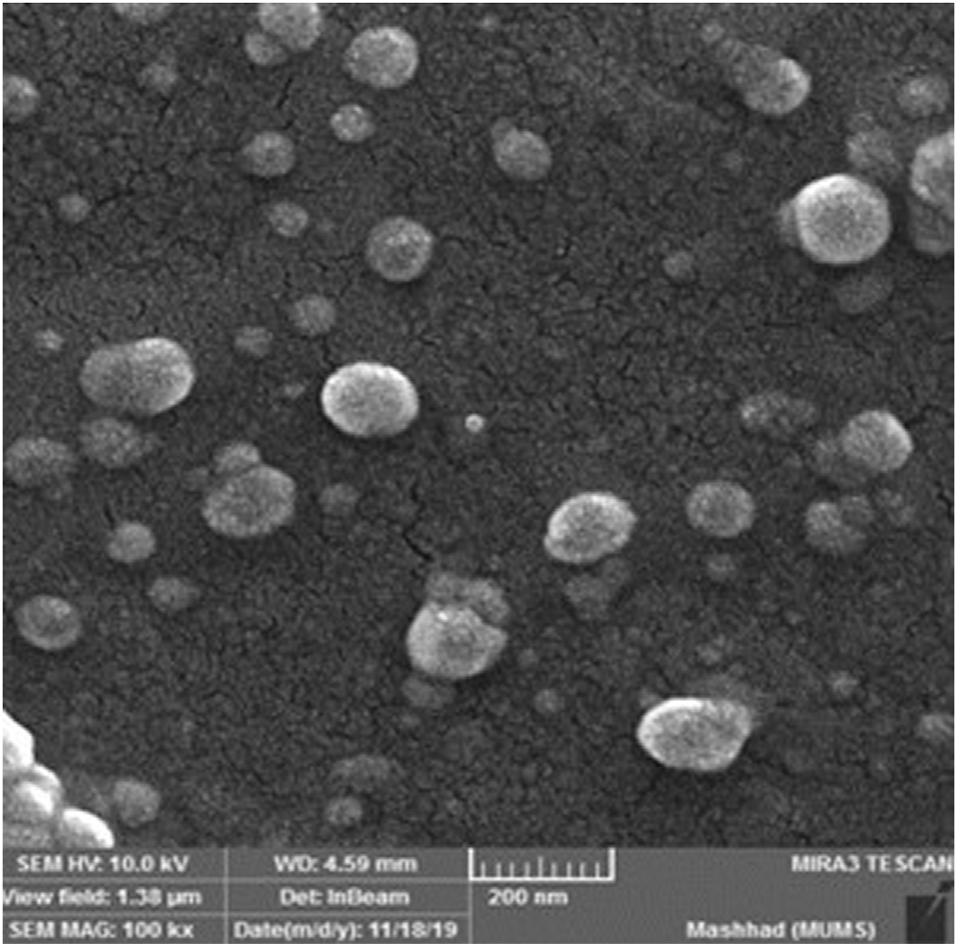
The FESEM analysis of *Achillea millefolium* phenolics-loaded nanoliposomes.

The results of particle size analysis indicated the nanometer size of the nanoliposomes (187.2 nm) and the PDI value of 0.213 lower than 0.3 revealed the homogeneous dispersion (Hasan et al., 2014). The zeta potential is a marker of the stability of colloidal dispersions and the potential value ranged from ±30 to ±40 mV is considered as a colloidal dispersion with moderate stability behavior (Figure 1). Thus, the colloidal dispersions of PRF-NLs showed moderate stability behavior. Finally, The microscopic analysis (Figure 2) confirmed the spherical shape of nanoliposomes. As well as homogeneous distribution of phenolic compounds.

### Phenolic Analysis of PRF-NLs

The total phenolic content of PRF-NLs was 7.04 ± 1.27 mg GAE/g DM. Meanwhile, the HPLC analysis illustrated different natural phenolic compounds such as gallic acid, syringic acid, ferulic acid, caffeic acid, ellagic acid, salicylic acid, and chrysin with the concentration ranging from 132 to 971 μg/g dried nanoliposomes (Table 3).

**TABLE 3:** Phenolic compounds presented in *Achillea millefolium* phenolics-loaded nanoliposomes.

Phenolic compounds (µg/g DW)						
GA	SY	FA	CA	EA	SA	CH
132 ± 3.1	305 ± 4.8	519 ± 4.6	832 ± 2.7	248 ± 3.3	971 ± 1.5	169 ± 3.8

GA, gallic acid; SY, syringic acid; FA, ferulic acid; CA, caffeic acid; EA, ellagic acid; SA, salicylic acid; CH, chrysin.

### Body Weight and Feed Intake Changes

Based on the results summarized in Table 4, the highest and lowest daily weight gain was observed in the mice group supplement with normal diet (T1) and infected group (T2), respectively with the values of 0.21 and 0.13 g/day. These results were consistent with the results obtained for average daily feed intake. Dietary addition of either non-encapsulated PRF or PRF-NLs at a concentration of 10 mg TPC/kg BW/day could improve the average daily and food intake significantly (*p* < 0.05). The improvement in the average daily weight gain and feed intake in the group of mice who received PRF-NLs was significantly (*p* < 0.05) higher than that of mice who received non-encapsulated PRF.

**TABLE 4:** The averages of mice body weight changes and feed intake upon receiving different treatments.

Average	T1	T2	T3	T4	SEM
Average daily weight gain (g)	0.21^a^	0.13^d^	0.15^c^	0.17^b^	0.05
Average daily feed intake (g)	3.4^a^	2.7^c^	3.1^b^	3.3^a^	0.11

T1: normal diet; T2: normal diet + infected by *C. jejuni* on day 21; T3: Normal diet enriched by PRF (10 mg TPC/kg BW/day) + infected by *C. jejuni* on day 21; T4: Normal diet enriched by nanoliposome-encapsulated PRF (10 mg TPC/kg BW/day) + infected by *C. jejuni* on day 21.

Values with diferent superscript letters in the same row are signifcantly diferent (p < 0.05).

### Liver Enzymes and Lipid Peroxidation

The biological markers of liver function and health such as ALT, SGPT, SGOT, and lipid peroxidation are presented in Table 5. As known, the increase in these parameters is the important symptoms of liver inflammatory and oxidative stress. Our results demonstrated that infection caused by *C. jejuni* notably (*p* < 0.05) elevated the liver enzymes and lipid peroxidation significantly. While the inclusion of non-encapsulated PRF and PRF-NLs in the dietary regimen of mice alleviated the liver enzymes and lipid peroxidation values significantly (*p* < 0.05). The modulation of liver enzymes and lipid peroxidation were more pronounce (*p* < 0.05) in the group of mice supplemented with PRF-NLs as compared to the mice that received non-encapsulated PRF.

**TABLE 5:** The results of liver enzymes and lipid peroxidation.

Liver enzymes	T1	T2	T3	T4	SEM
ALP (IU/L)	204.8^d^	258.1^a^	243.0^b^	228.7^c^	6.89
SGPT (IU/L)	98.5^d^	151.3^a^	139.4^b^	126.5^c^	3.57
SGOT (IU/L)	139.5^d^	173.5^a^	143.1^b^	135.6^c^	4.62
MDA (changes relative to control)	100^d^	132.5^a^	121.4^b^	116.7^c^	5.12

T1: normal diet; T2: normal diet + infected by *C. jejuni* on day 21; T3: Normal diet enriched by PRF (10 mg TPC/kg BW/day) + infected by *C. jejuni* on day 21; T4: Normal diet enriched by nanoliposome-encapsulated PRF (10 mg TPC/kg BW/day) + infected by *C. jejuni* on day 21.

Values with diferent superscript letters in the same row are signifcantly diferent (p < 0.05).

### Histopathological Evaluation and Morphometric Analysis

**FIGURE 3:**
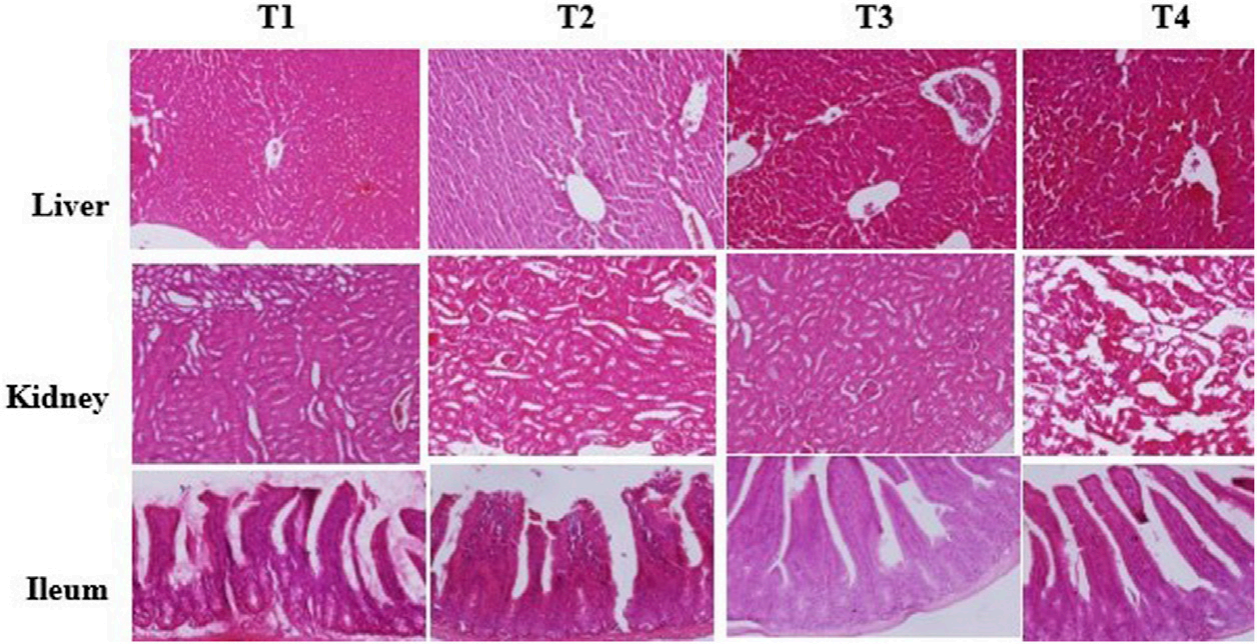
Histopathological analysis of liver, kidney, and ileum of the mice treated with different treatment. T1: normal diet; T2: normal diet + infected by *C. jejuni* on day 21; T3: Normal diet enriched by PRF (10 mg TPC/kg BW/day) + infected by *C. jejuni* on day 21; T4: Normal diet enriched by nanoliposome-encapsulated PRF (10 mg TPC/kg BW/day) + infected by *C. jejuni* on day 21.

The histopathological examination of liver, kidney, and ileum tissues is presented in Figure 3. The normal architecture was observed in the liver, kidney, and ileum tissues in the control group (T1). No prominent histopathological changes in the liver, kidney, and ileum tissues were observed upon infection by *C. jejuni* and supplementation of either non-encapsulated PRF or PRF-NLs. The morphometric analysis of ileum (Table 6) revealed that mice challenged by the infection caused by *C. jejuni* (T2) possessed higher crypt depth, villus with lower height, width, and decrease in the goblet cells quantity as compared to the control group (T1). The dietary addition of 10 mg TPC/kg BW/day from non-encapsulated PRF and nanoliposome-encapsulated PRF could significantly (*p* < 0.05) improve the morphostructural characteristics of the ileum. The improvement in the morphostructural characteristics of the intestine resulted in better absorption of nutrients and subsequently enhance the daily weight gain. These observations were consistent with the early study conducted by (Jamroz et al., 2006) who reported the stimulatory effects of plant bioactive compounds on growth and development of villus, increase in the production of mucus on the inner wall of the intestine which prevents enteropathogens colonization.

**TABLE 6:** Morphometric analysis of ileum in the mice receiving different treatments.

Parameters	T1	T2	T3	T4	SEM
Villus Height (µm)	423.8^c^	408.7^d^	435.4^b^	452.6^a^	7.86
Villus Width (µm)	127.4^c^	101.3^d^	138.2^b^	145.1^a^	9.34
Crypt Depth (µm)	132.5^b^	152.6^a^	134.3^b^	114.7^c^	7.66
Mean number of Goblet Cells	5.1^a^	3.5^b^	3.6^b^	4.7^a^	0.86

T1: normal diet; T2: normal diet + infected by *C. jejuni* on day 21; T3: Normal diet enriched by PRF (10 mg TPC/kg BW/day) + infected by *C. jejuni* on day 21; T4: Normal diet enriched by nanoliposome-encapsulated PRF (10 mg TPC/kg BW/day) + infected by *C. jejuni* on day 21.

Values with diferent superscript letters in the same row are signifcantly diferent (p < 0.05).

### Gene Expression Analysis

The expression analysis of inflammation- (COX2 and iNOS) and antioxidant-related (SOD and GPx) genes in the ileum tissue are presented in Table 7. The intestinal infection caused by *C. jejuni* significantly (*p* < 0.05) up-regulated the inflammation-related genes COX2 and iNOS by 5.1 and 6.8 folds, respectively as compared to the control (T1). In contrast, the *C. jejuni* infection down-regulated the expression of antioxidant-related genes (SOD, GPx) by 3.89 and 1.93 folds, respectively, as compared to the control group. The dietary supplementation of 10 mg TPC/kg BW/day from non-encapsulated PRF and nanoliposome-encapsulated PRF significantly (*p* < 0.05) suppressed the expression of inflammation- and increased the expression of antioxidant-related genes significantly (*p* < 0.05).

**TABLE 7:** Gene expression analysis of the mice received different treatments.

Fold changes					SEM
Genes	T1	T2	T3	T4	
COX2	1.0^d^	+5.1^a^	+3.3^b^	+2.4^c^	0.06
iNOS	1.0^d^	+6.8^a^	+4.7^b^	+3.3^c^	0.17
SOD	1.0^c^	−3.89^d^	+1.1^b^	+1.8^a^	0.09
GPx	1.0^c^	−1.93^d^	+1.8^b^	+2.1^a^	0.14

T1: normal diet; T2: normal diet + infected by *C. jejuni* on day 21; T3: Normal diet enriched by PRF (10 mg TPC/kg BW/day) + infected by *C. jejuni* on day 21; T4: Normal diet enriched by nanoliposome-encapsulated PRF (10 mg TPC/kg BW/day) + infected by *C. jejuni* on day 21.

Values with diferent superscript letters in the same row are signifcantly diferent (p < 0.05).

The antioxidant and anti-inflammatory potentials of gallic acid, syringic acid, ferulic acid, caffeic acid, ellagic acid, salicylic acid, and chrysin have been previously documented (Shahidi and Ambigaipalan 2015; Ambriz-Pérez et al., 2016). Hence, the regulation of inflammation- and antioxidant-related genes in the current study might be due to the presence of bioactive phenolics compounds in the non-encapsulated and nanoliposome-encapsulated PRF. Apart from that, the higher intestinal solubility, absorption of nanoliposome-encapsulated PRF resulted in significant (*p* < 0.05) down-regulation in the inflammation- and up-regulation in antioxidant-related genes as compared to the mice received non-encapsulated PRF.

### Relative Quantification of *Campylobacter* jejuni

**FIGURE 4:**
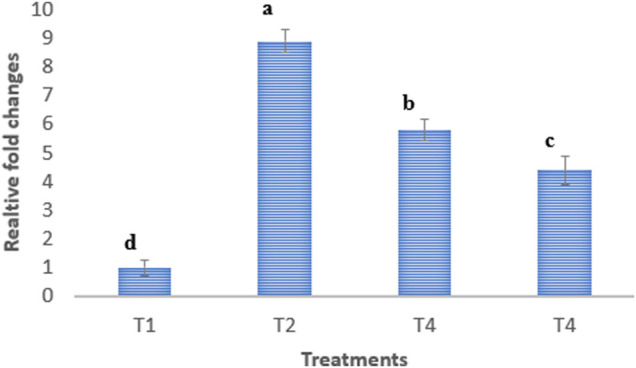
Relative fold changes of *C. jejuni* population in ileum digesta. T1: normal diet; T2: normal diet + infected by *C. jejuni* on day 21; T3: Normal diet enriched by PRF (10 mg TPC/kg BW/day) + infected by *C. jejuni* on day 21; T4: Normal diet enriched by nanoliposome-encapsulated PRF (10 mg TPC/kg BW/day) + infected by *C. jejuni* on day 21.

The relative changes in the ileal population of *C. jejuni* are presented in Figure 4. The results illustrated that challenge with *C. jejuni* significantly (*p* < 0.05) increased the population of *C. jejuni* in the ileum digesta by 8.9 folds as compared to the control un-infected group of mice.

The dietary supplementation of non-encapsulated and nanoliposome-encapsulated PRF notably (*p* < 0.05) suppressed the *C. jejuni* population in the ileum digesta by 5.8 and 4.4 folds, respectively. The inhibition of *C. jejuni* in the ileum by nan-encapsulated PRF and nanoliposome-encapsulated PRF is attributed to the presence of antibacterial compounds such as gallic acid, syringic acid, ferulic acid, caffeic acid, ellagic acid, salicylic acid, and chrysin (Bouarab-Chibane et al., 2019) in the PRF.

These results postulated that nanoliposome-encapsulated PRF more effectively modulated the enteropathogenic *C. jejuni* in the ileum as compared to the nonencapsulated PRF. The higher antibacterial activity of nanoliposome-encapsulated PRF as compared to the non-encapsulated PRF could be associated with the higher intestinal solubility and absorption of nanoliposome-encapsulated PRF. Thereby, the nanoliposome-encapsulated PRF could be considered as a natural antibiotic alternative called phytobiotic to prevent intestinal infection caused by *C. jejuni.* Besides, these bioactive phenolics present in the PRF, stimulate the production of intestinal mucus which then create a thick layer of mucus on the inner wall of the ileum and reduces the possible colonization of *C. jejuni* and resulted in the reduced population of *C. jejuni* (Jamroz et al., 2006).

## Conclusion

The nanoliposome-encapsulated PRF and non-encapsulated PRF at the concentration of 10 mg TPC/kg BW/day could improve the average daily weight gain, food intake, liver function, antioxidant status, and morphostructural characteristics of the ileum and decreased the ileum population of *C. jejuni* in the mice challenged by *C. jejuni* infection, however, the nanoliposome-encapsulated PRF was more functional effective than nonencapsulated PRF in alleviating the side effects of *C. jejuni* infection. Consequently, the nanoliposome-encapsulated PRF could be considered as a promising phytobiotic against *C. jejuni* infection in mice. For the future work we suggest the isolation and encapsulation of phenolics individually and determine the phytobiotic properties against *C. jejuni.*

## Data Availability

The raw data supporting the conclusion of this article will be made available by the authors, without undue reservation.
